# Personal

**Published:** 1917-03

**Authors:** 


### PERSONAL

Dr. F. William Welch of Buffalo was injured Feb. 1 by a collision between his auto and a lumber wagon, the tongue of which penetrated the side of the auto. He was confined to bed for five days but has fully recovered.

Dr. John T. Harris has succeeded Dr. Wm. Warren Britt as Health Officer of Tonawanda, at a salary of $800. Dr. Britt held the position for several years.

Dr. George E. Vincent, President of the University of Minnesota, has been elected President of the Rockefeller Foundation. He will assume office May 1, 1917, and will serve for three years.

Dr. R. M. Root of Buffalo left Feb. 9 to spend the rest of the winter in Florida.

Major N. G. Russell, M. D., of Buffalo, left Feb. 9 for a rest at Old Point Comfort.

Dr. Leon Hamilton was operated upon for appendicitis Feb. 10.

Dr. P. A. McCreary of East Aurora returns from Florida the middle of March.

The following Buffalo physicians returned with the 74th Regt. Feb. 20: Capt. Arthur C. Schaefer, First Lt. G. McKay Hall, First Lt. Carlton E. Wertz, First Sergeant A. C. Callahan.

